# Drug-Induced Cholestasis Secondary to Meropenem in an Elderly Patient: Report of A Rare Case

**DOI:** 10.7759/cureus.103145

**Published:** 2026-02-07

**Authors:** Abhishek Shrestha, Ahmed Suliman, Talat Bazrbachi, Sung-eun E Kim

**Affiliations:** 1 Internal Medicine, The Princess Alexandra NHS Trust, Harlow, GBR

**Keywords:** alkaline phosphatase (alp), carbapanem, drug induced cholestasis, drug induced hepatotoxicity, meropenem therapy

## Abstract

Meropenem is a broad-spectrum carbapenem antibiotic commonly used in hospitals to treat moderate-to-severe infections. Although rare, meropenem can cause drug-induced liver disease, including mild transaminase elevation, cholestasis, or hepatic failure. This report presents an 80-year-old woman admitted after a mechanical fall and diagnosed with a catheter-associated urinary tract infection. Intravenous meropenem was initiated following urine culture confirmation of Citrobacter koseri in the context of worsening confusion and elevated inflammatory markers. Six days after starting meropenem, the patient developed generalised weakness and mild pruritus. Liver function tests showed significant abnormalities: alkaline phosphatase (ALP) 1450 U/L and alanine transaminase (ALT) 415 U/L. Liver function improved after meropenem was discontinued. The pattern of injury was consistent with cholestatic drug-induced liver injury (DILI) (R-value = 1), and an updated Roussel Uclaf Causality Assessment Method (RUCAM) score of 8 indicated a probable causal relationship with meropenem.

This case highlights the importance of considering DILI, even with short-term antibiotic use, especially in elderly patients. Routine monitoring of liver function is recommended for those receiving meropenem. Early recognition and prompt withdrawal of the offending agent are essential for clinical improvement and prevention of complications.

## Introduction

Meropenem is a broad-spectrum carbapenem antibiotic with excellent potency against both Gram-positive and Gram-negative bacteria [1]. Carbapenems inhibit cell wall biosynthesis and are classified as bactericidal agents. Meropenem is effective in treating moderate to severe bacterial infections, including those caused by mixed or multidrug-resistant organisms [2]. Its broad-spectrum activity makes it suitable for serious hospital-acquired infections of unknown origin [3].

Carbapenems, including meropenem, are generally well-tolerated antibiotics, with the most common gastrointestinal adverse effects being nausea, vomiting, and diarrhoea. These agents are primarily excreted unchanged by the kidneys into the urine. Consequently, acute hepatotoxicity associated with meropenem is rare, likely due to minimal hepatic metabolism. Most carbapenems have been reported to cause transient, mild to moderate, and asymptomatic increases in aminotransferase levels, which typically resolve rapidly after discontinuation of therapy [4].

Drug-induced liver injury (DILI) is a rare yet clinically significant complication associated with meropenem therapy [5]. Cholestatic DILI may present with a spectrum of clinical manifestations, ranging from mild non-specific symptoms such as fatigue, fever, anorexia, weakness, vomiting, chills, right upper quadrant pain, pruritus, and skin rash to severe, prolonged jaundice, ascites, coagulopathy, and encephalopathy [6].

## Case presentation

An 80-year-old woman was admitted to the geriatric ward after recurrent falls and was diagnosed with a catheter-associated urinary tract infection. Her medical history included atrial fibrillation, congestive heart failure, hypertension, long-term catheterisation, and chronic venous insufficiency. Her regular medications were apixaban, colecalciferol, dapagliflozin, digoxin, famotidine, gabapentin, mebeverine, ropinirole, spironolactone, trimbow, and salbutamol inhalers, which she had been using long-term. Documented allergies were clarithromycin, bisoprolol, and perindopril. There was no history of liver disease or alcohol use.

Empirical treatment with nitrofurantoin 100 mg twice daily was initiated for the urinary tract infection. After three days, urine culture identified Citrobacter koseri, which was sensitive to nitrofurantoin, so treatment was continued. Despite the absence of sepsis, the patient developed confusion, and inflammatory markers increased, suggesting treatment failure. Consequently, nitrofurantoin was discontinued after four days.

As the isolated bacteria were resistant to penicillin and fluoroquinolones, including ciprofloxacin, intravenous meropenem 1 g every eight hours was initiated after consultation with the microbiology team, as the organism was sensitive to meropenem. Following initiation of meropenem, both confusion and inflammatory markers started to improve. On the sixth day of therapy, the patient developed new-onset generalised weakness and mild pruritus. The clinical examination revealed stable vital signs, no jaundice, and a normal abdominal examination with no tenderness or organomegaly. Baseline liver function tests were within normal limits at both admission and on the day meropenem therapy was initiated.

However, repeat testing six days after starting meropenem demonstrated significant abnormalities: elevated alanine aminotransferase (ALT) 415 IU/L (baseline 0-34 IU/L), elevated alkaline phosphate (ALP) 1450 IU/L (baseline 46-122 IU/L), elevated aspartate aminotransferase (AST) 322 IU/L (baseline 5-34 IU/L), and elevated gamma-glutamyl transferase (GGT) 1535 IU/L (baseline 0-38 IU/L). Total bilirubin was mildly elevated at 1.52 mg/dl (baseline 0.2-1.3 mg/dl). Other laboratory investigations, including complete blood count and renal function tests, remained normal. Intravenous meropenem was discontinued immediately due to suspected drug-induced liver injury. The ALT began to improve after cessation of meropenem. The ALP increased to 1827 IU/L the following day before subsequently declining. Both AST and GGT levels also improved. The patient's symptoms, including weakness and pruritus, resolved after discontinuation of meropenem.

**Figure 1:**
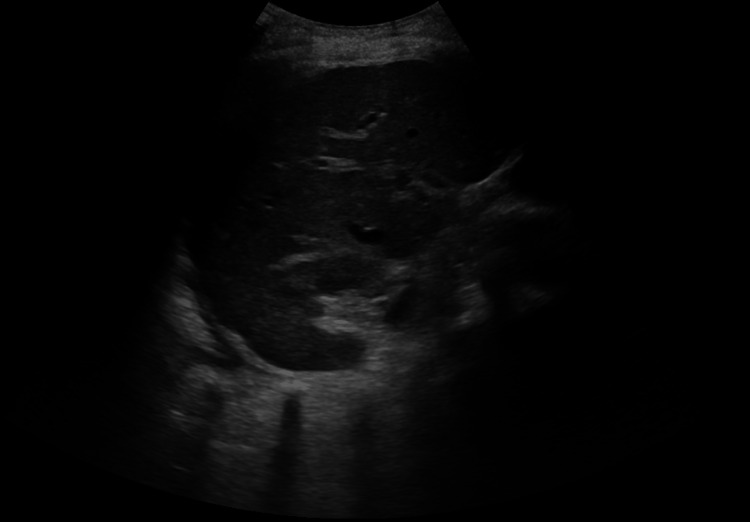
Liver ultrasound demonstrating normal echogenicity of liver without biliary dilatation

**Figure 2:**
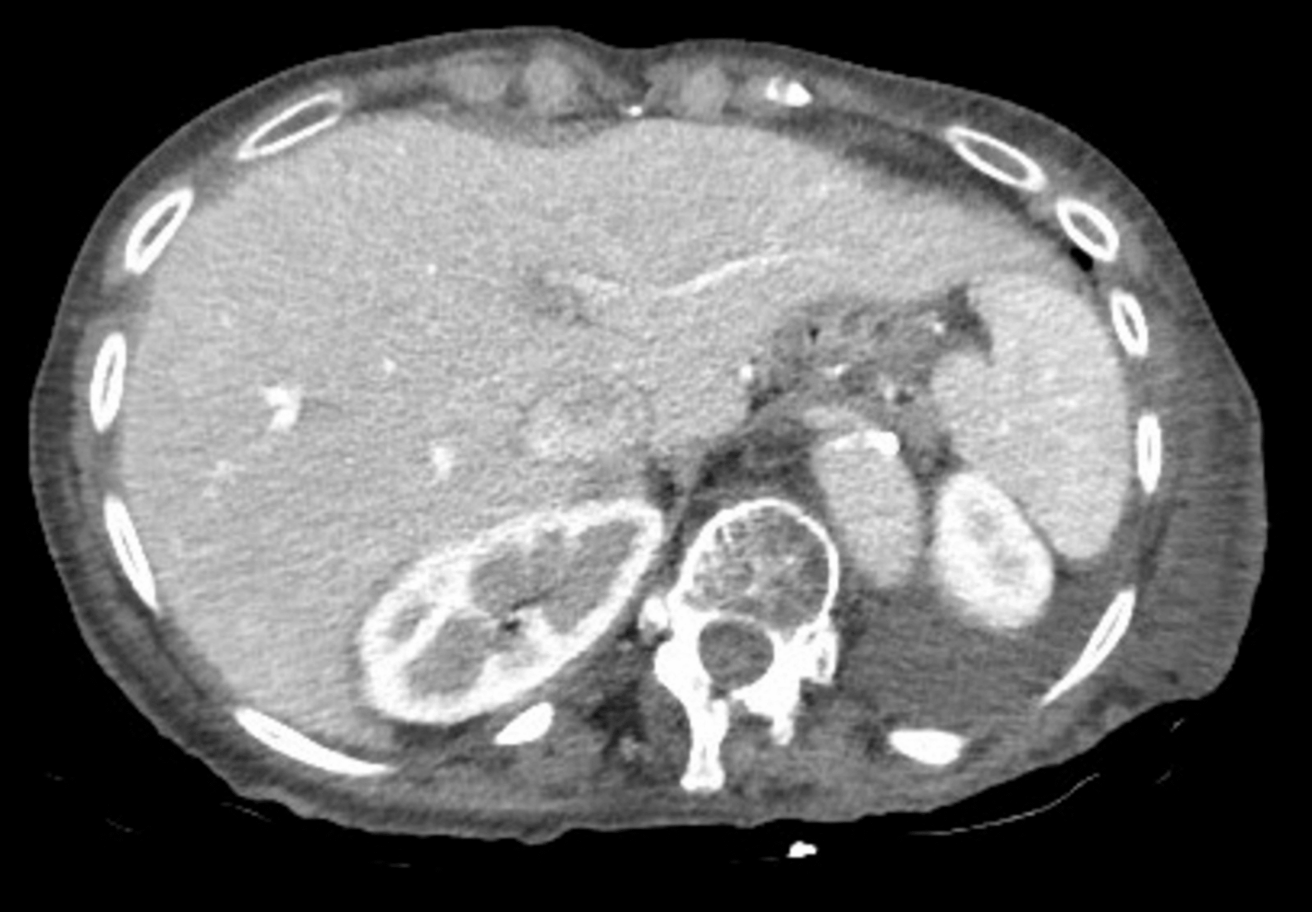
Contrast-enhanced CT of the abdomen and pelvis revealed no intrahepatic or common bile duct dilatation and a normal liver appearance.

The case was reviewed with the gastroenterology team, who concluded that the elevated liver function tests were most likely attributable to drug-induced cholestasis, based on normal baseline values, the temporal association with meropenem administration, and subsequent improvement after discontinuation. Additional blood tests and imaging were performed to exclude alternative causes of liver dysfunction. Hepatitis serology for types A, B, C, and E was negative, as were tests for cytomegalovirus and Epstein-Barr virus. Immunoglobulin levels and coagulation profile, including international normalised ratio (INR), were within normal limits. Liver ultrasound demonstrated normal echogenicity without biliary dilatation (Figure 1). Contrast-enhanced CT of the abdomen and pelvis revealed no intrahepatic or common bile duct dilatation and a normal liver appearance (Figure 2).

**Figure 3:**
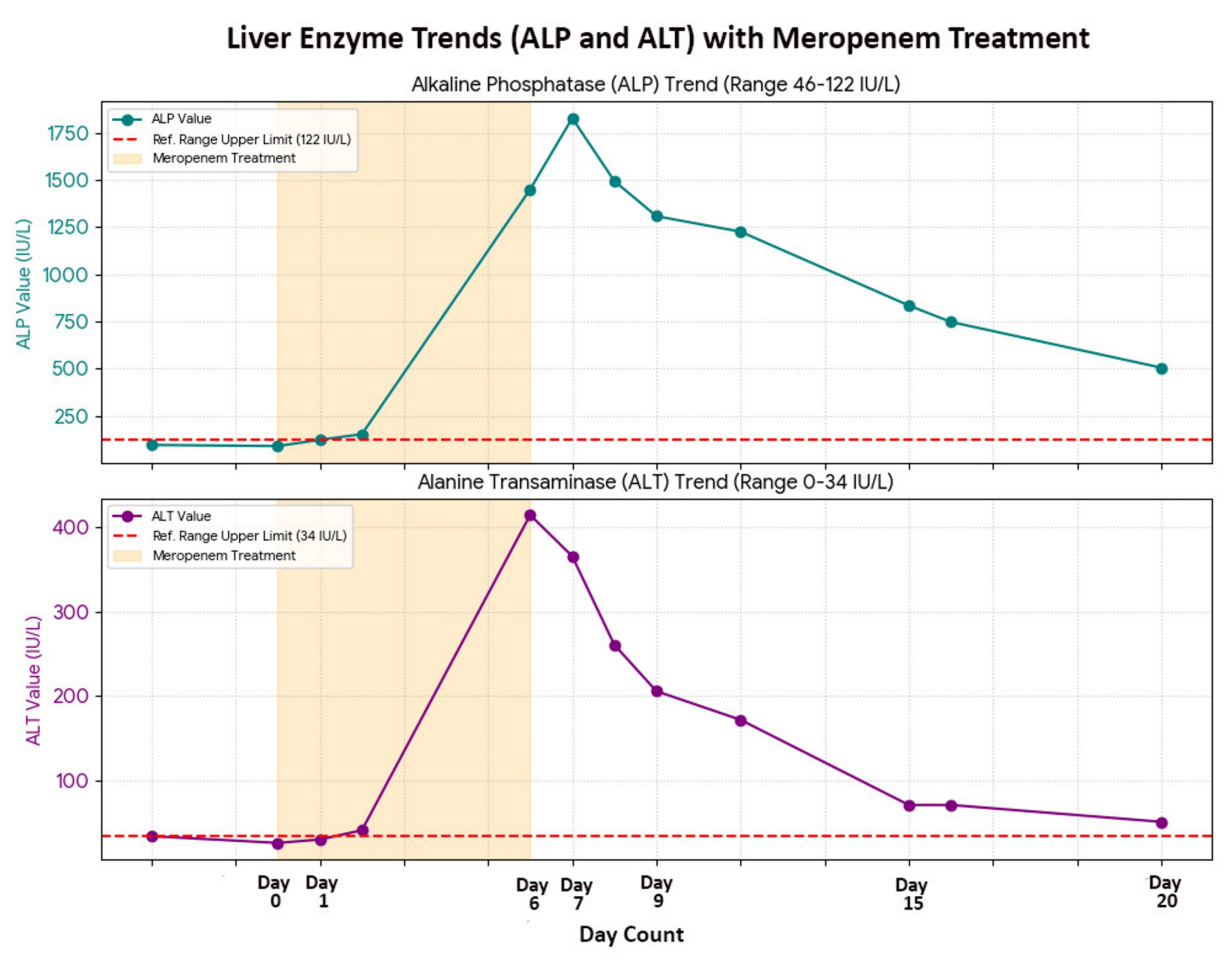
Timeline of liver enzymes trend (ALP and ALT) with meropenem therapy ALP: Alkaline phosphatase, ALT: Alanine transaminase

The autoimmune liver screen, including antinuclear antibody, mitochondrial antibody, smooth muscle antibody, gastric parietal cell antibody, antinuclear microsomal antibody, and thyroid peroxidase antibody, was negative. Twenty days after discontinuation of antibiotics, ALT decreased to 51 IU/L and ALP to 506 IU/L (Table 1) (Figure 3). The patient was discharged with instructions to avoid hepatotoxic medications and to follow up with her general practitioner for repeat liver function tests in 3 to 4 weeks.

**Table 1:** Trend of liver function tests with duration of meropenem therapy

Day of therapy	ALT (IU/L)	AST (IU/L)	Total bilirubin (µmol/L)	Alkaline phosphatase (IU/L)
Day 0	26	–	12	91
Day 6 (therapy stopped)	415	322	26	1450
Day 7	366	168	29	1827
Day 11	172	95	9	1226
Day 20	51	–	10	506

The pattern of liver injury in DILI was assessed using the R-value, calculated as the ratio of ALT to the upper limit of normal (ULN) divided by the ratio of ALP to the ULN. An R-value of 5 or greater indicates hepatocellular injury, 2 or less indicates cholestatic injury, and values between 2 and 5 suggest a mixed pattern [7]. In this case, the R-value was 1, consistent with cholestatic injury secondary to meropenem use.

The updated Roussel Uclaf Causality Assessment Method (RUCAM), a widely used and validated tool for assessing DILI, was applied to evaluate the probability of DILI. The total RUCAM score was 8, indicating a 'probable' relationship [8]. Given the RUCAM score and the improvement in liver function tests after discontinuation of meropenem, liver biopsy was not performed.

## Discussion

Drug-induced liver injury (DILI) is the most common cause of acute liver failure (ALF) in the Western world [9]. It is categorized as either dose-dependent (direct) or idiosyncratic (dose-independent) [7]. Idiosyncratic DILI constitutes an uncommon adverse event associated with the use of prescription or non-prescription medications. Among these, antibiotics are identified as the most frequent cause of idiosyncratic DILI [9].

Meropenem is generally well tolerated; however, liver injury, particularly cholestasis, is a rare adverse effect, with only a few published case reports to date. The pathophysiology of meropenem-induced cholestasis remains poorly understood. Proposed mechanisms include direct hepatocellular toxicity, idiosyncratic reactions, and hypersensitivity. Additional risk factors may include genetic predisposition, pre-existing liver disease, and concurrent use of other hepatotoxic medications [5]. A retrospective study of hospitalized patients receiving meropenem identified male sex, intensive care unit admission, gallbladder disease, elevated baseline ALP and GGT levels, and lower baseline platelet counts as risk factors for meropenem-induced liver injury [2].

Idiosyncratic DILI is a diagnosis of exclusion, established only after alternative causes of liver injury, such as viral hepatitis, autoimmune disease, immunologic conditions, biliary obstruction, and malignancy, have been ruled out [10]. In the absence of specific diagnostic tests for idiosyncratic DILI, diagnosis depends on the temporal association between drug administration and abnormal liver function tests, as well as the resolution of symptoms and liver enzyme abnormalities following cessation of the suspected medication [9,10].

Sepsis-induced cholestasis must be excluded when evaluating drug-induced cholestasis, as the administration of broad-spectrum antibiotics for sepsis can create a misleading impression of antibiotic-induced liver injury. In sepsis-induced cholestasis, conjugated hyperbilirubinemia typically ranges from 2 mg/dL to 10 mg/dL. Serum ALP is usually elevated but rarely exceeds two to three times the upper limit of normal, and serum aminotransferase concentrations are generally only moderately increased [11]. In this case, the absence of clinical features of sepsis, together with markedly elevated ALP and ALT but a bilirubin level less than 2 mg/dL, which occurred only after meropenem initiation, excluded sepsis-induced cholestasis.

The primary therapeutic intervention for suspected DILI is the immediate withdrawal of all potential causative agents and avoidance of rechallenge [5,7,9,10]. Early identification of cholestasis is critical, as prompt discontinuation of the offending agent can facilitate rapid recovery of liver function [5]. Close laboratory monitoring is necessary during the initial days and weeks following DILI detection, particularly in patients with pre-existing liver disease or those receiving hepatotoxic medications [5,7]. Clinicians should obtain baseline liver enzyme levels before initiating antibiotic therapy and monitor liver enzymes regularly throughout treatment [5]. The offending drug should also be documented in the patient's allergy or adverse event notes to prevent recurrence of DILI.

Initial management also involves symptomatic treatment and general supportive care, including the administration of antiemetics, analgesics, and parenteral hydration [7]. Corticosteroid therapy has a limited role in DILI and is reserved for cases involving immune-mediated DILI or drug-induced autoimmune hepatitis (AIH). In cases of acute liver failure, prompt referral to a liver transplantation unit is indicated [9].

In this case, the patient developed cholestatic liver injury, presenting with generalized weakness, mild pruritus, and abnormal liver enzyme levels following meropenem therapy. Early recognition of DILI enabled prompt discontinuation of meropenem, resulting in symptom resolution and improvement in liver enzyme levels.

## Conclusions

Meropenem is widely used in hospital settings to treat moderate-to-severe bacterial infections due to its broad-spectrum antimicrobial activity. While meropenem may cause mild, asymptomatic elevations in transaminase levels, it can, in rare cases, result in significant liver injury. Meropenem-induced liver injury is often underdiagnosed and overlooked, which contributes to its reported low incidence. This case report highlights the importance of considering drug-induced liver disease in the differential diagnosis of patients presenting with unexplained jaundice, pruritus, or elevated liver enzymes, particularly following recent antibiotic use. Additionally, this case illustrates that early recognition and prompt discontinuation of the causative antibiotic can facilitate clinical improvement and prevent complications of further hepatic injury.
